# Evaluation of subacute bisphenol – A toxicity on male reproductive system

**DOI:** 10.14202/vetworld.2015.738-744

**Published:** 2015-06-17

**Authors:** S. S. Karnam, R. C. Ghosh, S. Mondal, M. Mondal

**Affiliations:** 1 Department of Veterinary Pathology, College of Veterinary Science and Animal Husbandry, Chhattisgarh Kamdhenu Vishwavidyalaya, Durg, Chhattisgarh, India; 2 Department of Veterinary Pathology, West Bengal University of Animal and Fishery Sciences, Kolkata, West Bengal, India; 3 Department of Veterinary Pathology, College of Veterinary Science and Animal Husbandry, Nanaji Deshmukh Veterinary Science University Rewa, Madhya Pradesh, India

**Keywords:** bisphenol A, hematology, rat, spermatozoa, testes

## Abstract

**Aim:**

The aim was to evaluate the effect of multiple oral administration of bisphenol A (BPA) for 28 days on seminal characteristic on mammal using Wistar rat as a model.

**Materials and Methods:**

Rats were randomly divided into five different groups having 6 male rats in each group. The doses chosen were 50, 200, and 600 mg/kg body weight for Groups III, IV and V, respectively, based on preliminary dose range finding study and Group II served as vehicle control and Group I was negative control.

**Results:**

Reproductive study in the BPA-treated rats on day 28 revealed that there was significant (p≤0.05) reduction in the epididymal sperm count of rats of Group IV and significant (p≤0.01) decrease in Group V. Sperm motility percentage, dead count percentage, head and tail abnormality percentage were found to be significantly (p≤0.01) increased in rats of BPA-treated groups as compared to rats of control groups. Testes showed necrosis of germinal layer and spermatogonial cells in the seminiferous tubules. Hematological examination revealed significant (p≤0.01) decrease in the mean values of total erythrocyte count (TEC), total leukocyte count (TLC), hemoglobin, packed cell volume, and there was also significant (p≤0.05) lymphocytopenia in treated animals.

**Conclusion:**

It can be concluded from this study that subacute toxicity of BPA caused a reduction in the epididymal sperm count, sperm motility, dead count, head and tail abnormality, as well as hematological indices such as TLC, TEC etc. Hence, it appears that BPA affects the germ cells leading to impairment in the spermatogenesis, and thus having its property as reproductive toxicant and it also suppresses bone marrow functioning, which leads to normocytic hypochromic anemia in rats.

## Introduction

India is widely developing country, and its lifestyle is largely on a change. In this scenario, plastic is sufficiently being used in all spheres of life. Plastic has a lasting use, and it has proved in various known applications with wide public acceptance. At present, bisphenol A (BPA) is being used in the manufacturing of several types of plastics including polycarbonates, epoxy resins, and polyvinyl chloride [1].

The concern about potential exposure of BPA is based on reports indicating leaching of BPA from plastics [2-4] and food cans lined with epoxy resins [5]. BPA molecules linking with ester bond in polycarbonate and resins is subject to hydrolysis, resulting in leaching of BPA monomer even from polycarbonate and resins into the water at room temperature [6].

BPA leaches from dental composites and from food containers such as cans and polycarbonate plastic water bottles and the consumption, as well as production of plastic material, is increasing by the time and simultaneous exposure of BPA to animals, wildlife species, and humans is also increasing [7]. Recent discoveries regarding the environmental distribution and presence of BPA in animals, wildlife species, and humans have generated persistent scientific, regulatory, and public interest in assessing the potential health risks associated with BPA exposure. Endocrine disruptors cause adverse health effects in humans and wildlife species subsequent to changes in endocrine function. BPA is one of the chemicals identified as a potential endocrine disruptor based on its estrogenic properties and toxic effects on germinal cells, which lead to disturbance in hormone production also.

The present study was designed to evaluate the toxic effect in Wistar rat by evaluating semen after multiple exposures to BPA.

## Materials and Methods

### Ethical approval

Experimental protocol was approved by Institutional Animal Ethics Committee, College of Veterinary Science & Animal Husbandry, Anjora, Durg, Chhattisgarh under the guidance of Committee for the Purpose of the Control and Supervision of Experiments in Animals, Government of India, before starting the experiment.

### Animals

The present study was conducted on 6 weeks old healthy Wistar rats. The rats were procured and housed in cages at Animal House, College of Veterinary Science and Animal Husbandry (Chhattisgarh Kamdhenu Vishwavidyalaya), Anjora, Durg, Chhattisgarh, India. Animals were acclimatized to the experimental room for 7 days before the start of the experiment.

### Husbandry

The animals were housed in polypropylene cages under controlled environment and hygienic conditions. Animals were provided standard feed and allowed water *ad libitum* throughout the experimental period.

Chemicals: BPA was procured from S D Fine Chem, India.

### Formulation

BPA was formulated using propylene glycol as a vehicle. BPA solution was administered orally in Groups III-Vwith dose volume of 10 ml/kg. The daily oral administration was continued for 28 days.

### Experimental design

Experimental groups consist of five groups having 6 male rats each. Rats of Group I was kept as negative control and was given only distilled water orally. Rats of Group II served as vehicle control and were given propylene glycol. Rats of Group III-V were administered BPA, formulated in propylene glycol. Dose rate for Group III-V was 50, 200, and 600 mg/kg body weight, respectively, for 28 days. Doses tested in this experiment were based on primary dose range finding study.

### Sperm evaluation

At the end of the experiment, all the rats were sacrificed by using guillotine and then testicles were removed to collect epididymis for evaluation of sperm.

### Sperm count

Epididymal sperm collection and sperm counting were carried out according to the procedure suggested by Del Val and Robledano [8]. Cauda epididymis from rat was collected in petri-dish (Garg Process Glass India, Mumbai). It was minced with sharp scissors. Torned epididymis was transferred to a test tube containing 4 ml Dulbecco phosphate buffer saline (Gibco, catalog no- 14040-133) at 37°C temperature. Then the sperms were allowed to disperse for 5-10 min and sperm counts were made in the Neubauer’s chamber (Precision Scientific Instruments, Delhi) using a pipette. Sperms were observed at high power of the microscope.

### Sperm motility

Epididymal sperm motility was carried out as per the procedure suggested by Saalu *et al*. [9]. Sperm motility was also made by transferring a small amount of the diluted suspension on a pre-warmed slide and then applying a cover slip. Sperm motility was recorded in percentage (%).

### Sperm morphology

Sperm head morphology evaluated by the process adopted by Luke *et al*. [10]. Briefly, 1 ml of sperm suspension was transferred to a marked test tube. Then 5-6 drops of 1% Eosin yellow was added to it and gently mixed it by simple finger tapping. 45 min incubation was done in room temperature to facilitate the staining. Then the suspension was agitated gently by pipette. Smear was prepared by simple push technique. The smears were air-dried and preserved by mounting with cover slips on the following day using DPX mountant. About 100 sperms/rat from each experimental group was examined at high power.

### Live and dead sperm count

The microscopic identification of live and dead spermatozoa was carried out as per the method suggested by Correia *et al*. [11]. A drop of the epididymal sperm suspension was placed on one edge of the clean glass slide and mixed with a drop of 3% eosin using a glass rod. Immediately this mixture was again mixed with a drop of 10% nigrosin using the same glass rod. A small quantity of this was smeared on another slide and air-dried. 200 sperms of each rat were counted at high power and live and dead spermatozoa counts were recorded. The nigrosin stain produces a dark background on which the sperms stand out as lightly colored objects. Normal live sperms excluded the eosin stain and appeared whitish, whereas dead sperms lost membrane integrity, taken up eosin, and appeared pinkish in color.

### Hematological studies

After 28 days of treatment with BPA, before sacrificing the animal, blood was collected from retro-orbital plexus with the help of a capillary tube. Rats were anesthetized by using isoflurane (Baxter, USA). Ethylenediaminetetra acetic acid was used as an anticoagulant for hematological examinations (4 mg/ml). Thin blood smears were prepared for differential leukocyte count. Hematological studies were carried out on the day of collection of blood.

### Pathological study

The representative tissue samples were collected in 10% neutral buffered formalin and processed for histopathological studies. The routine protocol adopted at the Department of Veterinary Pathology, College of Veterinary Science and A. H., Anjora, Durg was employed for histopathological examination. Sections were cut at 3-5 μ and stained with hematoxylin and eosin as per the method described by Longo *et al*. [12].

### Statistical analysis

Statistical analysis was done using one-way ANOVA by using graph pad prism software (vesion-5) followed by Dunnett’s t-test to see the significance if any.

## Results

### Sperm evaluation: Sperm count, sperm motility, dead sperm count, abnormal head sperm, and abnormal tail sperm

In the present study, epididymal sperm evaluation e.g., epididymal sperm count (×106 sperms/ml), epididymal sperm motility (%), dead sperm count (%), abnormal head sperm (%), and abnormal tail sperm (%) in different groups of rats treated with BPA at different dose levels have been shown in Table-1.

**Table-1:** Effect of daily oral administration of bisphenol A on various sperm abnormalities in Wistar rats (n=6).

Group	Total sperm count (×10^6^/ml)	Motility (%)	Dead sperm (%)	head abnormality (%)	Tail abnormality (%)
Group I	31.66±1.45	63.33±1.20	21±0.57	3.33±0.88	7.33±0.33
Group II	31±2.08	63.66±0.33	20±1.52	3±0.57	7.66±0.66
Group III	28.66±0.88	59±1.15**	25.33±0.88**	7.66±0.66**	18.66±1.45**
Group IV	25.33±1.45*	47.33±0.33**	34.33±0.33**	12.33±0.88**	25.33±0.88**
Group V	21±1.15**	27±1.15**	45.33±1.20**	18.33±1.45**	32.3±1.45**

Superscripts may read column-wise for comparison of means.

* (p≤0.05) and

** (p≤0.01)

**Figure-1:**
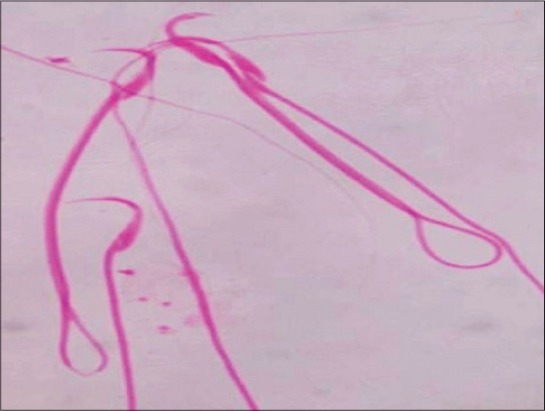
Photograph of the rat (Group V) showing coiled tail sperm (eosin stain ×2000).

**Figure-2:**
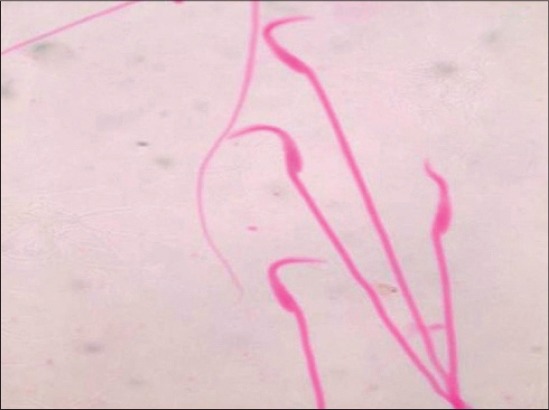
Photograph of the rat (Group V) showing banana shaped head sperm (eosin stain ×2000).

The administration of BPA orally for 28 days caused significant (p≤0.05) reduction in the epididymal sperm count of rats of both Groups IV and V. The mean values of sperm motility percentage were also decreased in dose-dependent manner. On the other hand, dead count percentage, head abnormality percentage and tail abnormality percentage were found to be significantly (p≤0.01) increased in rats of Groups III-V as compared to rats of control groups. Representative figures of live and dead sperm, abnormal tail sperm and abnormal head sperm are depicted in Figures-1 and 2.

### Hematological studies

In the present study, hematological parameters e.g., total erythrocyte count (TEC) (10^6^/µl), hemoglobin (Hb) (g/dl), packed cell volume (PCV) (%), mean corpuscular volume (MCV) (fl), mean corpuscular Hb (MCH) (pg), MCH concentration (MCHC) (g/dl), total leukocyte count (TLC) (10^3^/µl), and differential leukocytic count (%) in different groups of rats treated with BPA at different dose levels have been shown in Table-2.

**Table-2:** Effect of subacute bisphenol A toxicity on hematological parameters in Wistar rats (n=6).

Parameters	Groups				

	Group I	Group II	Group III	Group IV	Group V
Hb (g/dl)	16.26±0.44^c^	16.83±0.85^c^	15.33±0.51^bc^	14.46±0.35^b^**	11.13±0.37^a^**
PCV (%)	40±1.31^c^	43.5±1.17^d^	37.16±0.90^bc^	34.66±0.71^b^**	27.83±1.13^a^**
TEC (*10^6^/cu.mm)	6.35±0.25^b^	6.22±0.22^b^	5.94±0.26^b^	5.58±0.33^ab^	4.94±0.28^a^**
MCV (fl)	63.1±2.55^ab^	70.23±3.37^b^	63.26±3.94^ab^	63.28±4.26^ab^	56.21±3.6^a^
MCH (pg)	25.63±0.96^a^	27±1.01^a^	25.81±0.57^a^	26.33±1.65^a^	22.45±1.41^a^
MCHC (g/dl)	40.86±1.88^a^	38.71±2.01^a^	41.47±2.13^a^	41.69±0.66^a^	40.06±0.67^a^
TLC (*10^3^/cu.mm)	6.69±0.20^c^	6.79±0.23^c^	6.34±0.19^bc^	5.98±0.09^b^**	5.42±0.14^a^**
Lymphocyte (%)	76.67±0.98^c^	76.17±0.70^bc^	72±2.09^b^	67.5±1.31^a^*	64.83±1.70^a^*
Neutrophil (%)	21.83±0.65^a^	22.5±0.61^ab^	24.5±1.83^abc^	28±2.08^c^*	27.33±2.55^bc^*
Monocyte (%)	1.5±0.22^a^	1.33±0.21^a^	2.16±0.30^ab^	2.83±0.54^ab^	2.33±0.33^ab^
Eosinophil (%)	0.5±0.22^a^	0.5±0.22^a^	0.66±0.21^a^	1.33±0.42^a^	1.5±0.42^a^
Basophil (%)	00.00^a^	00.00^a^	00.00^a^	0.16±0.16^a^	0.33±0.21^a^

Superscripts may read row-wise for comparison of means. Similar superscripts showing means do not differ significantly.

* (p≤0.05) and

** (p≤0.01), Values indicate mean±SE,

SE=Standard error, TEC=Total erythrocyte count, TLC=Total leukocyte count, MCV=Mean corpuscular volume, MCH=Mean corpuscular hemoglobin, Hb=Hemoglobin, PCV=Packed cell volume, MCHC=Mean corpuscular hemoglobin concentration

The administration of BPA orally for 28 days caused significant (p≤0.01) reduction in the levels of Hb, PCV, and TLC of rats belonging to Groups IV and V compared to rats of control group (Groups I and II). There were no significant (p≤0.05) differences in MCV, MCH, and MCHC in rats of all the five groups. The mean values of TEC were found to be significantly (p≤0.01) decreased in rats of Group V as compared to rats of control groups (Groups I and II). Significant (p≤0.05) increase in the mean values of percent neutrophil was found in the rats of Groups IV and V as compared to rats of control groups (Groups I and II). However, there was a significant decrease (p≤0.05) in percent lymphocyte count in the rats of Groups IV and V as compared to rats of control groups (Groups I and II). There were no significant differences on percent monocyte, eosinophil, and basophil in BPA administered rats as compared to rats of control groups.

### Histopathology of testes

**Figure-3:**
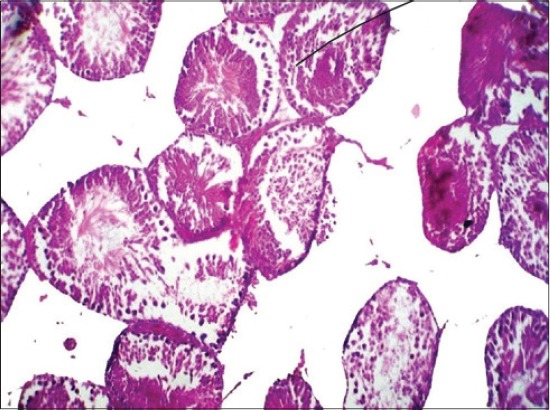
Section of testes of the rat (Group V) showing severe degenerative and necrotic changes in seminiferous tubules (H and E ×100).

**Figure-4:**
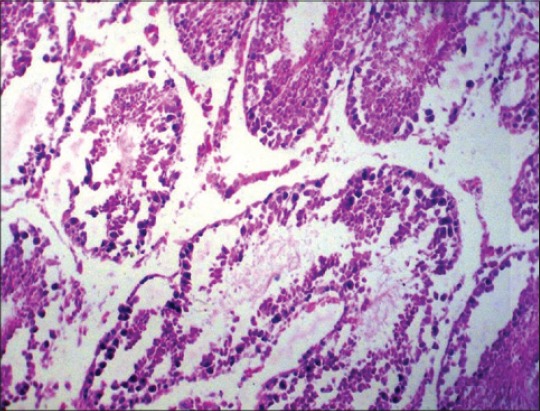
Section of testes of the rat (Group IV) showing severe degenerative and necrotic changes along with loss of spermatogonial cells (H and E ×100).

**Figure-5:**
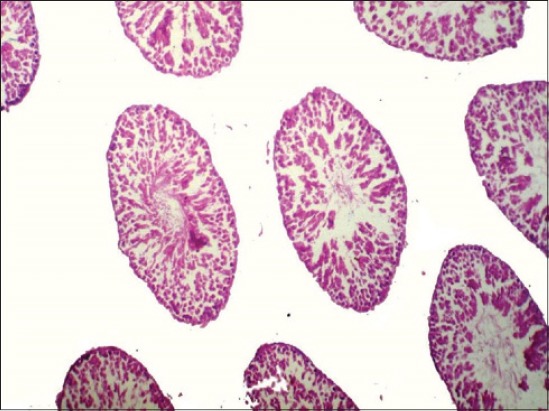
Section of testes of the rat (Group III) showing mild degenerative and necrotic changes (H and E ×100).

No appreciable gross lesions were seen in testes of all the experimental groups. Severity of histopathological changes in the testes of BPA-treated rats was dose dependent. Histopathological lesions in testes in Group V were moderate, included necrosis of germinal layers, and spermatogonial cells in the seminiferous tubules (Figure-3). In Group IV, the testes revealed mild degenerative and necrotic changes of germinal layers and spermatogonial cells in the seminiferous tubules (Figure-4). In Group III, the testes also revealed very mild degenerative changes (Figure-5).

## Discussion

Our findings are in accordance with the findings of Kourouma *et al*. [13]. According to them reduction in the epididymal sperm quality and sperm count were found in a dose-dependent manner when BPA was administered at dose rate of 2, 10, and 50 mg/kg body weight per day by intraperitoneal injection for 20 days in adult rats. Reduced epididymal sperm motility, sperm count, and abnormal sperm were associated with the augmented dose of BPA [14-16]. Even at very low doses some worker found that BPA decreased sperm count [17-21]. Some studies demonstrated low sperm count when BPA administered at the dose 200 mg/kg daily for 28 days [21-23]. BPA as noted in this present study directly affect spermatogenesis and testicular damage [24].

Studies focusing on exposure to environmental factors (pesticides, phthalates, PCBs, air pollution, trihalomethanes, mobile phones) on semen quality by some worker revealed low sperm concentration, sperm motility, and also abnormal sperm morphology [25,26].

Motility is a necessary function for sperm transport through the male and female reproductive tract. In this study, motility was significantly reduced in the BPA-treated groups compared with control groups, suggesting that BPA might have altered the mitochondrion, producing delay in motility and in some cases, eventually leading to sperm death resulting in reduced total sperm count [26,27]. The explanation given for the reduced total sperm count and reduced sperm motility were again possible reasons for increased dead sperm counts due to BPA toxicity in the present study.

There is evidence indicating that the development of the normal sperm head was polygenically controlled, and the increased numbers of sperm with abnormalities had reflected the genotoxic effects of prooxidants on germ cells [28]. The increased abnormalities of sperm observed in this study suggested that BPA might lead to genotoxic damage in germ cells. A more severe level of BPA elicited higher levels of abnormal sperm, showing a dose-dependent response. Clegg *et al*. [29] noted several sources of sperm morphological alterations *viz*. mutagenic events, non-mutagenic events or cellular degeneration, etc. Even in the absence of a complete understanding of the mechanism of abnormality induction, however, it appeared that the assay of sperm abnormalities would be of value. The male gametes may be examined rapidly. It was opined that, the agent, which induces sperm abnormalities was interfering either with the integrity of the DNA itself or with the expression of this genetic material. Detached heads and coiled tails were typical spermatozoa defects seen in testicular degeneration [30].

In the present findings, the significant decrease in sperm count and sperm motility along with an increase in the frequency of abnormal head sperm in the medium dose group and high dose group were the consistent findings. BPA produces spermatotoxic effect through induced alteration in testicular DNA and sperm chromatin structure [31]. This concept of pesticide inflicted sperm injury may be supported by the high positive correlation observed between mutagenicity and spermatotoxic effects [32]. Germ cell depletion from seminiferous tubules leads to reduced sperm production and abnormal sperm production. It was well supported by the present findings of testicular histopathology. Lemasters and Selevan [33] reported an alteration in sperm count and motility were all strongly and significantly associated with fertility in animals. Our results also suggested that BPA affected normal growth and development of sperm, resulting in decreased sperm motility and numbers, which might consequently contribute to the reduced fertility.

Therefore, our results suggested that BPA might have potential mutagenic effects on germ cells that led to abnormal sperm production.

Dose-dependent reduction in the diameter of the seminiferous tubule and reduced number of sertoli cells in the present study was in well agreement with data reported by Kim *et al*. [34] in mice. Studies [35,36] reported decreased the diameter of the seminiferous tubules together with the number of sertoli cell. Awal *et al*. [37] also observed decreased seminiferous tubule diameter when treated with BPA in male Wistar rats compared to those of control group. It was observed morphologically multinucleated giant cells having >3 nuclei in seminiferous tubules of testes following 13 weeks BPA treatment [38]. However, the exact mechanism of action of BPA as reproductive toxicant is still need to be studied [39].

Our findings are in accordance with the findings of Furukawa *et al*. (1994) who observed decrease in number of erythrocytes, Hb concentration and hematocrit values when induced a 13 weeks subchronic toxicity of BPA in male and female B6C3F1 mice at dose levels of 0, 0.2, 0.5, 1.0, 2.0, and 4.0% in the diet [40]. Matsumoto *et al*. [41] also observed slight microcytic anemia in Wistar rats of either sex, fed diet containing 0, 30, 100, 300 or 1000 ppm of 2, 4, 6-tri-tert-butylphenol for up to 24 months. Tilak *et al*. [42] recorded decrease in erythrocyte count, hemoglobin content and PVC in Indian major carps, *Catla catla, Labeo rohita*, and *Cirrhinus mrigala* after exposure to sublethal concentrations of phenol.

Woo *et al*. [43] conducted a 28 day repeated oral dose toxicity study of nonylphenol in Sprague-Dawley rats at doses of 0 (control), 10, 50 or 250 mg/kg body weight and observed development of anemia at 250 mg/kg in both sexes. Santa *et al*. [44] reported hemolytic anemia and mild hepatic injury in 21 years old healthy male due to accidental ingestion of phenol.

In the present study, the increase in dose of BPA and a simultaneous decrease in erythrocyte count indicated that the BPA might have toxic inhibition of bone marrow leading to reduced erythropoiesis resulting in normocytic hypochromic anemia. This study also showed that BPA caused a significant decrease in the levels of total leukocytes count. It has been suggested that compounds having benzene ring or other ring structure acts as a hapten that combines with a protein constituent of leukocytes to form an antigen to which animal develops antibodies, which are toxic to leukocytes causing either lysis or agglutination [45]. BPA is also a ring structure compound and thus may have caused leukocytopenia. It was noted that BPA caused a significant decrease in the level of lymphocyte in all treated groups. Continuous exposure to BPA might then lead to lymphocytopenia, which could have an immunosuppressive effect. It was very difficult to decide in the present case that the neutrophilia noticed in this experiment should be categorized as shift to left of schilling index or shift to right of schilling index as the number of immature neutrophils were very few. Therefore, the increase in neutrophils in the present experiment may be attributed partly to the stress the experimental animals were under and partly to the moderate inflammatory changes noticed in the various organs due to BPA toxicity [46]. It is important to note here that the low values of lymphocyte count in the present case do not represent as absolute lymphocytopenia, but in true sense, it is in fact the relative decrease in the lymphocyte count as there was absolute neutrophilia recorded due to both stress as well as the inflammatory response exhibited toward various organ damage caused by the BPA toxicity [45].

## Conclusion

In the present study of BPA induced subacute toxicity in wistar rats revealed that there was significant (p≤0.05) reduction in the epididymal sperm count in 200 mg/kg and 600 mg/kg dose group. Sperm motility percentage, dead count percentage, head and tail abnormality percentage were found to be significantly (p≤0.01) increased in rats of BPA-treated groups as compared to rats of control groups. Histopathological findings in the testes showed necrosis of germinal layer and spermatogonial cells in the seminiferous tubules. Moreover, results of hematological analysis preliminary indicates suppression of bone marrow in which target cells are germinal (both myeloid and erythroid) in nature, which are labile of BPA toxicity and stop to produce daughter cells. Thus, it appears that BPA affects the germ cells leading to impairment in the spermatogenesis.

## Author’s Contributions

This study is the major component of the work toward the M.V.Sc. thesis of SSK, under the guidance of the second author RCG. SSK conducted the experimental trials and organized the manuscript and RCG thoroughly revised the same. SM and MM were responsible for the housing and management of rats and helped in organ collection, data analysis and interpretation. All authors read and approved the final manuscript.
